# Subcutaneous Implantable Cardioverter Defibrillator: A Contemporary Overview

**DOI:** 10.3390/life13081652

**Published:** 2023-07-28

**Authors:** Fabrizio Guarracini, Alberto Preda, Eleonora Bonvicini, Alessio Coser, Marta Martin, Silvia Quintarelli, Lorenzo Gigli, Matteo Baroni, Sara Vargiu, Marisa Varrenti, Giovanni Battista Forleo, Patrizio Mazzone, Roberto Bonmassari, Massimiliano Marini, Andrea Droghetti

**Affiliations:** 1 Department of Cardiology, S. Chiara Hospital, 38122 Trento, Italy; eleonorabonvicini@yahoo.it (E.B.); alessio.coser@apss.tn.it (A.C.); marta.martin@apss.tn.it (M.M.); silvia.quintarelli@apss.tn.it (S.Q.); roberto.bonmassari@apss.tn.it (R.B.); massimiliano.marini@apss.tn.it (M.M.); 2 Electrophysiology Unit, Cardio-Thoraco-Vascular Department, ASST Grande Ospedale Metropolitano Niguarda, 20162 Milan, Italy; preda.alberto@hsr.it (A.P.); lorenzo.gigli@ospedaleniguarda.it (L.G.); matteo.baroni@ospedaleniguarda.it (M.B.); sara.vargiu@ospedaleniguarda.it (S.V.); marisa.varrenti@ospedaleniguarda.it (M.V.); patrizio.mazzone@ospedaleniguarda.it (P.M.); 3 Department of Thoracic Surgery, Candiolo Cancer Institute, FPO-IRCCS, Candiolo, 10060 Turin, Italy; forleo@me.com; 4 Cardiology Unit, Luigi Sacco University Hospital, 20157 Milan, Italy; adroghetti@libero.it

**Keywords:** subcutaneous implantable cardioverter defibrillator, ventricular tachycardia, sudden death, cardiomyopathy

## Abstract

The difference between subcutaneous implantable cardioverter defibrillators (S-ICDs) and transvenous ICDs (TV-ICDs) concerns a whole extra thoracic implantation, including a defibrillator coil and pulse generator, without endovascular components. The improved safety profile has allowed the S-ICD to be rapidly taken up, especially among younger patients. Reports of its role in different cardiac diseases at high risk of SCD such as hypertrophic and arrhythmic cardiomyopathies, as well as channelopathies, is increasing. S-ICDs show comparable efficacy, reliability, and safety outcomes compared to TV-ICD. However, some technical issues (i.e., the inability to perform anti-bradycardia pacing) strongly limit the employment of S-ICDs. Therefore, it still remains only an alternative to the traditional ICD thus far. This review aims to provide a contemporary overview of the role of S-ICDs compared to TV-ICDs in clinical practice, including technical aspects regarding device manufacture and implantation techniques. Newer outlooks and future perspectives of S-ICDs are also brought up to date.

## 1. Introduction

The development of S-ICDs from concept to their initial commercialization was a journey lasting 19 years. Only in 2009 and 2012 did the first generation of S-ICDs receive the CE mark and US FDA approval, respectively. The S-ICD was developed as a possible alternative to transvenous ICDs (TV-ICDs), trying to achieve the same effectiveness as TV-ICDs in terms of detecting and treating both ventricular fibrillation (VF) and ventricular tachycardia (VT) [1,2]. Several studies were performed in order to evaluate the efficacy and safety of these devices and rapid advances were made in the following years, leading to the development of a second generation of S-ICDs in 2015 and a third generation in 2016.

S-ICDs are structurally similar to TV-ICDs, being made of a pulse generator and a defibrillator coil. The advantage of S-ICDs concerns the components, which are completely outside of the chest. This substantial difference minimizes the risk of lead fractures or systemic infections, some of the most feared complications of TV-ICDs [3], as well as making any extraction procedure much simpler and less dangerous [4]. Consequently, the outlook for S-ICDs is stronger in two scenarios: when used in younger patients, who are usually affected by genetic heart diseases and are at high risk of sudden cardiac death (SCD) such as hypertrophic cardiomyopathy (HCM), dilated cardiomyopathy (DCM), and genetic arrhythmia syndromes [5,6,7]; and in instances in which the transvenous route is inaccessible. Nevertheless, S-ICDs present several limitations compared to TV-ICDs: due to the lack of an endocardial electrode, S-ICDs are only able to deliver post-shock ventricular pacing for 30 s. For this reason, for patients who need anti-bradycardia pacing or resynchronization therapy, S-ICD implants are contraindicated [8]. Another issue concerns the alloy of which the coil is composed, which contains a small amount of nickel (around 16%). However, the device is registered as nickel free and no cases of allergic reactions have been reported in allergic patients so far.

In recent years, larger studies confirmed the role of S-ICDs as a valuable alternative to TV-ICDs (Table 1). In both prospective trials [9,10,11,12] and registries [13,14], S-ICDs showed remarkable safety in the short and medium term, which was associated with a relatively low inappropriate shock rate in populations with different clinical characteristics and cardiovascular diseases, as well as indications of primary or secondary prevention of SCD. In this review, we provide an overview of the current role of S-ICDs in clinical practice compared to TV-ICDs, as well as updates to surgical techniques, medical management, and future perspectives of this increasingly used technology.

## 2. Subcutaneous ICD: What We Know So Far

### 2.1. Pre-Implant Screening

S-ICDs consists of a completely extra-thoracic device without the registration of intracardiac electrograms. For this reason, when a S-ICD implant is planned, it is necessary to ensure optimal sensing through a pre-implant screening [16]. The pre-implant screening aims to evaluate the amplitude of the sensed R wave and if the available three sensing vectors (primary from the proximal electrode ring to can, secondary from the distal electrode ring to can, and the third from the distal to the proximal electrode) are able to differentiate the R wave from the T wave in order to ensure appropriate sensing of VT and avoid inappropriate ICD shocks (IAS) [17]. The electrogram analyzed by the S-ICD is more similar to a standard 12-lead electrocardiogram (ECG) than to an intracavitary electrogram, with a distinct P-wave, T-wave and QRS-complex. A dedicated tool is used to measure the amplitude of the three sensing vectors from the standard 12-lead ECG in both a supine and a sitting/standing position. The screening is passed if at least one of the vectors works in both positions. Different studies demonstrated that 8% to 15% of the individuals are excluded from the implant of S-ICD after the screening [18,19,20]. Because many IAS are observed during exercise, some studies have suggested the possibility of conducting the screening during exercise to evaluate the three vectors in a dynamic way [21,22]. The most frequent cause of IAS in implanted S-ICD is T waves oversensing; therefore, in such cases, prolonged screening periods and a more detailed study of the T variation in different contexts are needed to improve the screening phase [23,24]. Exercise screening should be recommended in specific diseases with higher incidence of screening failure, such as HCM [25].

### 2.2. Implant Technique

The implant of S-ICD differs from a TV-ICD. S-ICD is made of a case pulse generator that is placed in a subcutaneous pocket between the anterior and the mid-axillary lines at the level of the V-VI intercostal space. Currently, a third-generation S-ICD device provided by Boston Scientific (EMBLEM; Boston Scientific, Marlborough, MA, USA) is used. It weighs 130 g and it measures 83.1 × 69.1 × 12.7 mm. It is magnetic resonance (MRI) compatible.

There is a single 45 cm lead with sensing ring electrodes at its extremities. One extremity is tunneled in the subcutaneous plane from the case to the sternum, where it is fixed 1 cm cranial to the xiphoid process while the other extremity is rounded and tunneled vertically parallel to the left side of the sternum.

To optimize the implant, different techniques have been tested. The first cases used a three-incision technique with two incisions at the extremities, one for the lead and one for the case. After that, a two-incision technique was developed using just the inferior incision for the placement of the lead and eliminating the superior one. Several studies demonstrated that the two-incision technique is as safe and efficacious as the three-incision one, providing a faster and less complicated procedure [26,27]. A high probability of effective defibrillation with a two-incision procedure was also reported [28].

Regarding the placement of the pulse generator, different sites of implant were evaluated. An intermuscular implant in the virtual space between the anterior surface of the serratus anterior muscle and the posterior surface of the latissimus dorsi muscle was demonstrated to reduce the risk of infections [29]. This technique could be also useful when insufficient subcutaneous tissue is available, such as in thin patients with a low body mass index or for cosmetic reasons [30]. In one study, the intermuscular implant reduced the shock impedance in obese patients [31]. Finally, a sub-serratus implant, by reducing the distance between the generator and the heart, may improve device efficacy and provide a better cosmetic effect, but only a few studies of this nature have been conducted [32].

Fluoroscopy is not necessary during an S-ICD implant, except in the pre-procedural step when finding the landmarks used for implantation. The procedure is mainly performed under deep sedation or general anesthesia [13] and the total duration of the procedure is demonstrated to be just a little longer than that of the transvenous one [27].

The S-ICD implant has a lower rate of severe complications compared to TV-ICD. Despite a slightly higher frequency of pocket hematoma, it strongly reduces the risk of pneumothorax, traumatic pericardial effusion, and lead dislodgment, with lower rates of re-intervention [27]. In the IDE study, no cases of cardiac perforation, tamponade, pneumothorax, or subclavian vein stenosis were registered [9].

The implant technique has been improved over the last 10 years of experience. In particular, it has been demonstrated that there is a steep learning curve for physicians who perform S-ICD implants, with only around 13 implants needed to acquire good autonomy. Increased experience with implantation techniques also led to a significant reduction in complication rates [33].

### 2.3. Inappropriate Shocks

ICD shocks are potentially associated with myocardial injury, altered hemodynamic, apoptosis, and inflammatory signaling [34]. Several studies demonstrated a positive relation between the burden of ICD shocks and development or worsening of heart failure, as well as increased risk of heart failure hospitalizations and mortality [35,36,37]. Moreover, shocks have non-negligible psychological and physical impact on patients, with the risk of seriously affecting their quality of life for decades [38]. Older studies reported that up to 17% of people with TV-ICD could receive an IAS, usually due to misinterpretation of supraventricular tachycardias (SVT), including sinus tachycardia, atrial fibrillation (AF), and atrial flutter or device malfunction [39,40]. This issue has been appreciated a lot in recent years and led to the development of newer optimized and focused diagnostic strategies, which progressively lessened the rate of IAS over time up to 1.9%, according to recent studies [41]. Regarding S-ICD, inappropriate T oversensing and myopotentials are the main cause of IAS [42,43]. On the contrary, S-ICD’s performance in discriminating AF seems higher than TV-ICD, according to a recent metanalysis [44]. In the IDE study, IAS was performed in 13.1% [15], while in the EFFORTLESS registry it was performed in 11.7% of cases, in addition to 2.3% of cases involving non-recognized SVT [13]. A more recent post approval study stated that 6.5% of cases involved IAS [14].

In the START study, the S-ICD algorithm was found to be effective for SVT discrimination, even better than TV-ICD [16]. Initial devices used single zone programming that was only capable of monitoring the cardiac rate. Improvements were made with the development of a second zone capable of conditional discrimination for rates between 170–240 beats/min. This zone is programmed to recognize rate and differentiate between SVT and VT with the possibility of achieving early diagnosis of AF. Dual zone programming strongly demonstrated a reduction in IAS incidence (11.7% vs. 20.5%) compared to single-zone programming [13,14].

The UNTOUCHED study reported the lowest rate of IAS for SVT among S-ICD controlled trials, with an IAS-free rate of 95.9% (*p* < 0.001) at 18 months (against a standard performance goal of 91.6% of TV-ICDs) [10]. Data from the UNTOUCHED study greatly differed from the data of the PRAETORIAN TRIAL [11], which reported higher rate of IAS in the S-ICD group, despite the absence of statistical significance. The reason for this discrepancy may be due to the higher prevalence of the third-generation S-ICD in the UNTOUCHED group compared to the PRAETORIAN one. Indeed, among the most important innovations of third-generation S-ICDs was the introduction of the SMART PASS filter (since 2018), which was designed to reduce the amplitude of lower-frequency signals (such as T-waves), maintaining unchanged signals from R-waves, VT or VF [45]. The introduction of SMART PASS effectively reduced the rate of IAS in another study [46]. This highlights the importance of morphology discrimination algorithms applied in the conditional shock zone in reducing IAS in S-ICDs as opposed to the initial use of interval criteria before applying morphology criteria in TV-ICDs [47].

### 2.4. Infections

The S-ICD Post Approval Study examined by Gold and colleagues [48] in order to evaluate the incidence and predictors of infections in a 3-year follow-up period observed an infection prevalence of 3.3% (69% within 90 days, 92.7% within 1 year, and none after 2 years). No lead extraction was needed. The mortality rate was 0.6%/year with no systemic infections. The results were similar to those of other previous studies.

Several meta-analyses reported no significant differences in the occurrence of device-related infections (OR = 1.57; 95% CI: 0.67–3.68) compared to TV-ICDs [49,50]. According to these data, the rates of all types of infection are the same between S-ICDs and TV-ICDs. However, a more accurate analysis identified a greater rate of high-risk infections (i.e., systemic infections) in the TV-ICD group. On the contrary, the S-ICD group was more prone to pocket infections, which are associated with a significantly lower risk of death [51].

In both cases, device removal is needed, although extractions of TV-ICD are significantly harder and have a higher risk of severe complications compared to S-ICDs extractions [52,53]. Patients at high risk of infection, such as dialyzed or immunocompromised patients, could benefit from S-ICD.

### 2.5. Lead Complications

Transvenous leads are the weakest elements of the TV-ICD system, causing dislocation, fracture, or infections. Lead fracture accounted for the first case of abandoned lead in the population with cardiac implantable electronic devices (CIEDs) [54]. The term “lead fracture” refers to a fracture in the lead’s conductor coil and typically accounts for less than 2% of IAS per year [55]. The risk increases in younger people and in females and becomes greater over time [56]. Lead fractures often occur in correspondence with stress points, such as near the pulse generator, at the venous access site, or at the lead tip, where repetitive motion places stress on the conductor coil. Lead fracture or displacement are often investigated when loss of sensing or pacing are detected during routine checks of the device. In ICDs, lead fractures are among the most frequent causes of IAS due to artifacts oversensing [57]. Moreover, a fracture of the high-voltage conductor coil may compromise the ability to deliver therapy when needed. In most cases of lead fracture, lead interrogation will show an increase in lead impedance, which may arise slowly or abruptly. Transvenous leads complications also include new or worsened tricuspid regurgitation, pericardial effusion or pericarditis, cardiac perforation with or without tamponade, hemothorax/pneumothorax, and upper-extremity vein thrombosis [58].

These conditions must be taken into account when a new device is implanted, especially in young individuals. New prospects have been offered by the S-ICD for this population, mainly due to the significant reduction in lead-related complications. In the PRAETORIAN trial, the primary endpoint consisted of a composite endpoint of device-related complications or inappropriate shocks at 4 years. The occurrence of lead-related complications was significantly higher in TV-ICD patients (6.6% in the TV-ICD arm versus 1.4% in the S-ICD arm; *p* = 0.001) [11]. The ATLAS trial reported 4.8% lead complications in the TV-ICD group compared to 0.6% in the S-ICD group at six months [12].

A recent meta-analysis conducted by Fong et al. substantially confirmed these data [44]. In particular, despite a similar rate of whole complications between the two groups (RR, 0.59 [95% CI, 0.33–1.04]; *p* = 0.070), a significant drop in the lead-related complications was found in the S-ICD group (RR, 0.14 [95% CI, 0.07–0.29]; *p* < 0.0001).

It is worth noticing that S-ICD lead-related complications are different from the ones of the TV-ICD groups because of the different conformation and position (Table 2). Indeed, the most frequent S-ICD lead-related complications happened in the early post-implant phase, consisting of lead movement and suboptimal lead position that usually only needed to be repositioned [49].

Data on long-term complications are still needed to perform a comprehensive comparison between the two devices.

### 2.6. Appropriate Therapies

The S-ICD has a reproducible good capacity for detection of VAs. In the IDE study, all VAs were successfully converted, with the exception of a self-interrupted monomorphic VT [15]. Similar data have been registered in the post-approval study, where only 5.3% of patients showed a VA with a conversion rate of 100% [9].

The START trial systematically compared the discrimination capacities between S-ICD and TV-ICD. In particular, at the end of S-ICD or TV-ICD implant, a VT was simulated and an appropriate detection rate (>99%) was registered in both groups [16]. On the contrary, in the PRAETORIAN trial, Knops et al. reported a higher rate of appropriate shocks in the S-ICD group. This result can be easily explained by the lack of S-ICDs to provide an anti-tachycardia pacing (ATP) therapy [59]. It must be considered that in the 4-year follow up of the PRAETORIAN trial, a switch from S-ICD to a TV-ICD was reported in 0.9% of cases. The reason was the need for anti-bradycardia pacing (0.7%) and the need for ATP therapy (0.2%) [11].

In conclusion, the efficacy of shock therapy was evaluated, with similar results between the two groups. The first shock efficacy was 93.8% in the S-ICD group and 91.6% in the TV-ICD group (*p* = 0.40) while efficacy of the last shock was 97.9% and 98.4%, respectively (*p* = 0.70) [59]. Accordingly, a 98% successful conversion rate was registered by Bardy and colleagues in one of the first observational studies [2].

S-ICD can deliver up to five consecutive biphasic shocks. The recharge lasts 14 s. The shock polarity can vary from coil to generator (standard) or generator to coil (reverse). The system is able to keep the last effective one in its memory. In cases of failure, the system automatically switches to an alternative mode. S-ICDs have a higher defibrillation threshold compared to TV-ICDs and deliver a biphasic shock of 80 J (versus 40 J of TV-ICDs). A study showed a lower increase in myocardial injury biomarkers in patients with S-ICD compared to TV-ICD after shock delivery [60].

## 3. Indications for S-ICD Implant

The current AHA/ACC/HRS guidelines (2017) indicate, in class I, the implant of S-ICD in patients who meet the criteria for an ICD when a high risk of infection or inadequate vascular access is present, but only if there is no expected need for anti-bradycardia pacing, cardiac resynchronization therapy, or VAs termination [61]. Instead, the latest ESC guidelines (2022) give the same indication with a IIa level of evidence.

According to current guidelines, the major reason to withhold an S-ICD implantation is the need for pacing. The need for anti-bradycardia pacing and cardiac resynchronization therapy (CRT) is the most frequent reason for excluding S-ICD. However, the inability of S-ICD to deliver ATP therapy is another non-negligible concern. Indeed, the history of monomorphic VT or non-sustained ventricular tachycardia (NSVT) increases the chance of appropriate ATP therapy in 1 out of 10 patients and 1 out of 3 patients, respectively [62]. This condition may be considered among all discriminant factors in the choice between S-ICD and TV-ICD. Alternatively, in cases of monomorphic VT or NSVT and contraindications for TV-ICD, the coupling of the S-ICD implant and VT ablation was shown to reduce the need of ATP [63].

Along with anti-bradycardia pacing, the battery longevity is another matter of concern when a S-ICD is considered. The first generation of S-ICD had a median battery longevity of 5 years [64] and, despite recent advances, the longevity of the third-generation S-ICD remains shorter than TV-ICD, with a median of 6 years.

In addition, S-ICD is incapable of direct sensing of atrial arrythmia and, although it features the possibility of remote monitoring, the data broadcast is not automated, but patient induced. Another limitation of S-ICD regards the cost, which is significantly higher than TV-ICD.

Despite the above-mentioned limitations, the implant of S-ICD should be considered as the first choice for a specific group of patients. In children and young people, S-ICD is safe and effective [65] and could be useful due to these patients’ long life expectancy and more active lifestyle. Indeed, transvenous lead-related complications are reported to be very high in this population and have been linked to a relevant risk of IASs [66]. In the ATLAS trial, which enrolled younger patients compared to other studies, a lower rate of major lead-related complication in S-ICD patients was noted [12]. Although there might be a mismatch between the size of the generator case compared to the available anatomical location in the lateral axilla in infants and small children, subcutaneous leads are better suited to body growth changes and therefore more adaptable for young people who are still growing.

Patients on dialysis may be also good candidates for S-ICD due to limited vascular access or partial obstruction of central veins, as well as the higher risk of systemic infections and complications related to the extraction [67,68]. Koman et al. reported similar procedural outcomes and inappropriate and appropriate shocks in hemodialysis patients with S-ICD compared to a control TV-ICD group [69].

**Figure 1:**
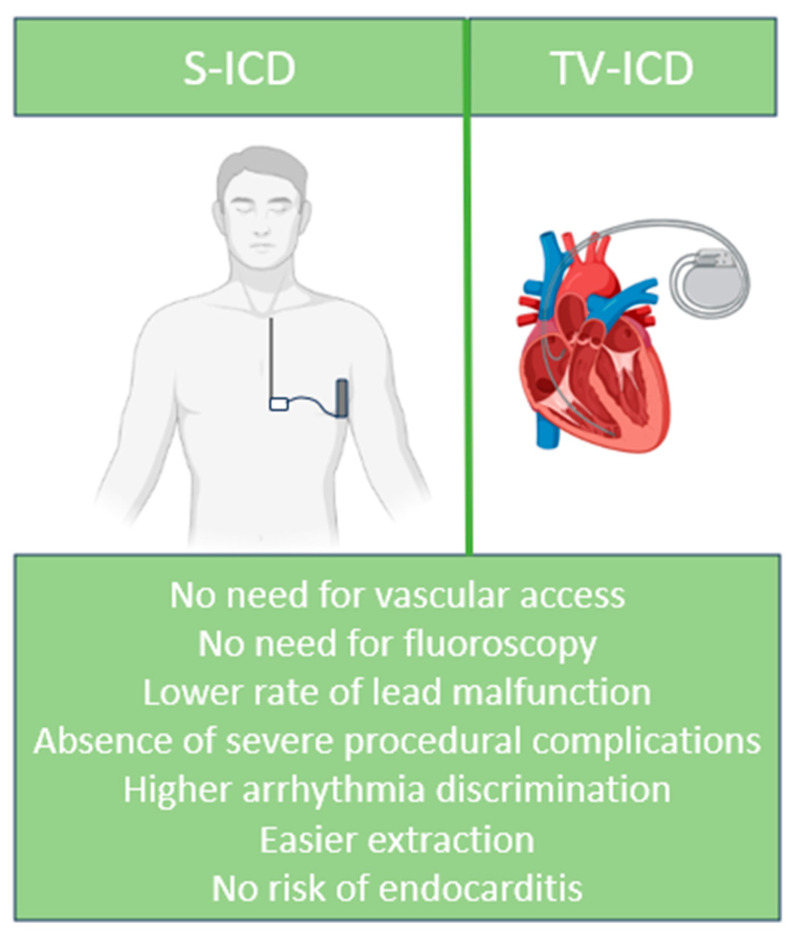
Advantages of S-ICD over TV-ICD.

In conclusion, patients at high risk of infections may benefit from a subcutaneous device: this group include patients with a previous device infection, patients with renal disease, and patients who are chronically immunosuppressed [63,68]. Figure 1 summarizes the advantages of S-ICD over TV-ICD.

## 4. Particular Set of Patients according to the Underlying Cardiomyopathy

### 4.1. Hypertrophic Cardiomyopathy

Patients with hypertrophic cardiomyopathy (HCM) may need ICD therapy from adolescence. As previously explained, since the risk of lead-related complications increases with age, S-ICD could be a good option in these patients [70]. The majority of HCM patients were found to be eligible for S-ICD implant after the screening step [71] and the safety and efficacy of S-ICD in HCM patients was similar to that of TV-ICD with a similar rate of post operative complications (92.7% vs. 89.5%), final shock conversion efficacy (100% vs. 98%), and IAS (12.5% vs. 10.3%, mainly due to T wave oversensing) [72]. Anti-bradycardia pacing is rarely needed in individuals affected by HCM. In cases of advanced disease or severe left ventricle outflow tract obstruction without other therapeutic opportunities, an intravenous device could be useful for resynchronization therapy or to reduce the outflow tract obstruction [7]. Notably, according to one study, patients with HCM and S-ICD had significantly fewer ICD interventions (due to an inability to deliver ATP) than patients with TV-ICD but without differences in shock delivery rate and mortality at follow-up [72,73]. These data suggest that among all ATP therapies delivered from the TV-ICD, a consistent number may be potentially unnecessary.

### 4.2. Brugada Syndrome, Long QT Syndrome and Arrhythmogenic Right Ventricular Cardiomyopathy

Young people with channelopathies such as Brugada syndrome (BrS) and long QT syndrome (LQTS) or those affected by genetic cardiomyopathies such as arrhythmogenic right ventricular cardiomyopathy (ARVC) may be good candidates for S-ICD due to its high safety profile [74,75]. However, while BrS and LQTS patients are not strictly dependent on ATP therapy due to more frequent incidence of polymorphic VTs [76,77], in arrhythmogenic cardiomyopathies the choice between a TV-ICD or S-ICD should be evaluated case by case through integration of clinical and genetic data [78]. Indeed, several arrhythmogenic cardiomyopathies including ARVC may have significantly higher incidence of sustained monomorphic TVs [79]. Consequently, this category of patients could have greater benefit from a device capable of delivering ATP therapies. Another non-negligible concern of ARVC is related to its evolutionary nature, leading to reduction in myocardial voltages over time and subsequent risk of IAS due to non-cardiac oversensing [80]. Therefore, a careful assessment of the risk–benefit ratio in this category of young individuals with higher risk of SCD should be performed, considering the higher risk of lead-related complications as well as the risk of an increase in the sensing and pacing threshold [81]. In BrS, the typical morphology of the ST tract could lead to oversensing on the T wave, interfering with the functioning of the device [82]. In these cases, careful screening is needed.

### 4.3. Congenital Heart Disease

Several studies have documented the safety and feasibility of S-ICD implantation in patients with congenital heart disease (CHD) or with vascular abnormalities [83,84]. An analysis of IDE study and EFFORTLESS registry showed similar rates of complications in the CHD versus the non-CHD group (10.5% vs. 9.6% [*p* = 0.89]) as well as IAS (10.5% vs. 10.9% [*p* = 0.96]) [83]. However, in this category of patients with higher rates of both SVT and VT, as well as macroscopic anatomical alterations, more studies are needed.

## 5. Future Perspectives

The use of S-ICD is rapidly spreading, particularly due to the absence of transvenous leads in this procedure and its significantly lower risk of long-term complications such as lead fracture and infections. However, several pitfalls must be considered and are summarized in Table 3. Of these, the most important limitation of the device is its inability to provide anti-bradycardia pacing or ATP. To overcome these limitations, the association of S-ICD with leadless pacemakers (with or without ATP capabilities) was proposed as an alternative to TV-ICDs. Only one case report of a combined implantation of S-ICD and a leadless pacemaker (without ATP capabilities) has been reported in human models [85]. According to the study, the two implants were safe even if not used at the same time. The authors declared no risk of oversensing detected by the S-ICD even with the maximum pacing output. Moreover, no concern about the potential for leadless pacemaker dysfunction after delivery of an S-ICD shock was reported and no device interactions were noted.

A second generation of leadless pacemakers capable of delivering ATP has been designed to work in combination with S-ICD. This association between two devices (EMPOWER™ Modular Pacing System and EMBLEM™ S-ICD [Boston Scientific, St. Paul, MN, USA]), known as the Modular CRM (mCRM) therapy system, avoids transvenous leads while providing the option to pace or deliver ATP. Some preclinical studies have been already conducted to test the correct device–device communication, as well as the ability to perform correct sensing, right ventricular stimulation, and ATP, reporting encouraging results even in the long term [86,87,88]. To further explore the safety, performance, and effectiveness of mCRM, the MODULAR ATP study was designed and started (ClinicalTrials.gov Identifier: NCT04798768).

Another strategy designed to integrate ATP therapy in non-TV-ICDs consists of placing a substernal lead in contact with the pericardium on top of the right ventricle, allowing for registration of direct cardiac signal [89]. So far, the only device with this characteristic available in commerce is the Extravascular-ICD Aurora (Medtronic). After Tung et al. successfully implanted a substernal lead in three patients for the first time [89], additional cases were published [90]. In the ASD2 study, the pacing, sensing, and defibrillating capability of a substernal lead was studied in 79 patients; ventricular pacing was effective in 97.4% patients, with a defibrillation threshold of 30 J needed to terminate 104 out of 128 episodes (81.3%) of VF [91]. Although only a few complications have been reported, tunneling the lead under the sternum requires extensive practice. More studies are needed to evaluate the long-term implications.

Unlike that of TV-ICD, the test of the defibrillation threshold (DFT) is still indicated after every S-ICD implant [92]. However, DFT testing is not without risks, as DFT testing-related death, stroke and prolonged resuscitation have been reported in small series [93]. For this reason, the possibility of a DFT-free implant is being considered. In a study involving 1290 patients, DFT performance was not associated with significant differences in cardiovascular mortality and ineffective shocks, suggesting that its omission may be safe [94]. The PRAETORIAN score is an algorithm developed to identify patients with high defibrillation thresholds using a routine chest radiograph and provides feedback to implanters on S-ICD positioning. The score is calculated by integrating the distance between the device and the thoracic wall and the distance between the device and the midline [95]. A low PRAETORIAN score means a low risk of conversion failure. The ongoing randomized PRAETORIAN DFT trial (ClinicalTrials.gov Identifier: NCT03495297) will evaluate avoidance of the DFT testing in well-positioned S-ICD [96].

The automatization of the remote monitoring transmission of CIEDs is of paramount importance and must be considered in future perspectives due to its crucial role in early interventions [97]. At present, remote transmission happens only by pressing a button on the receiver and not when an alert is registered. A reduction in patients’ compliance during follow up is reported [98].

Finally, results are expected from the 8-year follow up of the PRAETORIAN trial (PRATERORIAN XL). The primary objective is to establish the superiority of S-ICDs to TV-ICDs regarding acute and chronic complications.

## 6. Conclusions

S-ICD has proven to be safe and effective compared to TV-ICD. Innovations in pre-implant screening, implant technique, programming algorithm, and monitoring have already been developed, but further improvements are needed. New alternatives to overcome the limitations of S-ICD are in development. So far, S-ICD seems to be a valuable alternative to TV-ICD in some specific cases, such as those of young people or those with difficult vascular access, a high risk of infection, and no need for anti-bradycardia pacing or ATP.

## Figures and Tables

**Table 1:** Major studies on S-ICD.

Study	Year	Type	Aim of Study	Primary Endpoints	Secondary Endpoints	Results
IDE (Investigational Device Exemption) Trial [15]	2013	Prospective, non-randomized, multicenter clinical study	Safety and effectiveness of S-ICD	-Shock effectiveness in converting induced VF in conversion test -Complication-free Rate at 180 days	//	-100% VF conversion rate at 180 days -92–99% complications-free rate at 180 days
EFFORTLESS (Evaluation of factors impacting clinical outcome and cost effectiveness of the S-ICD) Registry [13]	2017	Prospective, non-randomized, multicenter observational registry	Early, mid- and long-term clinical effectiveness	-Complication-free rate at 30 days -Complication-free rate at 360 days -Inappropriate shocks-free rate for AF/SVT	//	-97% complication-free rate at 30 days -94% complication-free rate at 360 days -7% inappropriate shock rate (94% oversensed episodes)
S-ICD post approval Study [14]	2017	Prospective, non-randomized, multicenter registry	Safety and effectiveness of S-ICD	-Complication-free rate at 60 months -Shock effectiveness in converting spontaneous VT/VF at 60 months	-Electrode-related complications-free rate at 60 months -First shock effectiveness i converting induced and spontaneous VT/VF at 60 months	-96.2% complication- free rate at 30 days -98.7% successful conversion rate of induced VT/VF at 60 months
PRAETORIAN (Prospective randomized comparison of subcutaneous and transvenous implantable cardioverter defibrillator therapy) Study [11]	2020	Prospective, randomized, international, controlled trial	Comparison of safety and effectiveness in TV-ICD and S-ICD (non-inferiority)	-Adverse event rate at 48 months	-MACE, appropriate and inappropriate shocks, time to successful therapy, first shock conversion efficacy, implant procedure time, hospitalization rate, fluoroscopy time, cardiac (pre)-syncope events, cross over to the other arm, cardiac decompensation at 48 months -Quality of life at 30 months	-No difference in overall and arrhythmic mortality -Four times lead-related complications rate in TV -ICD -Two times infection rate in TV-ICD -No difference in complications rate in 4 years -No difference in inappropriate shock rate
UNTOUCHED (Understanding outcomes with the S-ICD in primary prevention patients with low ejection fraction) Study [10]	2021	Prospective, non-randomized, multinational trial	Safety and effectiveness of S-ICD	-Inappropriate shocks free rate at 18 months	-Freedom from system and procedure related complication at 30 days -All cause shock free rate at 18 months	-95.9% inappropriate shock-free rate at 18 months -90.6% all-cause shock-free rate at 18 months -92.7% complications-free rate at 18 months
ATLAS (Avoid transvenous leads in appropriate subjects) Trial [12]	2022	Prospective, randomized, multicenter controlled study	Comparison of safety and effectiveness in TV-ICD and S-ICD (superiority)	-Lead-related complications at 6 months -Other complications at 6 months	-Late device-related complications after 6 months -Arrhythmic deaths, visits, inappropriate shocks, all-cause mortality, economic analysis, patients acceptance after 6 months	-12 times lead-related complications in TV-ICD

**Table 2:** Transvenous ICD vs. subcutaneous ICD.

	TV-ICD	S-ICD
Pre-implant Screening	Not needed	Needed
Implant Technique	Transvenous	Subcutaneous
Sedation	Local	Deep/general anesthesia
Fluoroscopy	Needed	Not needed
Electrocardiogram	Intracavitary ECG	12-lead ECG
Inappropriate shocks	SVT	T oversensing, myopotential, discrimination error
Anti-tachycardia pacing	Possible	Not possible
SHOCK threshold	5–30 J	80 J
Infections	Systemic infections	Pocket infections
Lead complications	Dislocations/fractures; tricuspid regurgitation, pericardial effusion or pericarditis, cardiac perforation	Lead movement/suboptimal lead position

**Table 3:** Indications and pitfalls of S-ICD.

Indications	Pitfalls
Indication for ICD when pacing for bradycardia, cardiac resynchronization or ATP is not needed	Disease progression with need for anti-bradycardia pacing, cardiac resynchronization or enhancement of antiarrhythmic medical therapy
Congenital heart disease	Frequent development of conduction system disfunction overtime
Anatomical barriers to IV-ICD implantation (i.e., venous occlusion)	Aesthetic defect in thin women
History of IV lead infection	Only defibrillation therapy provided
Immunocompromised individuals	Large surgical wound, need for large disinfection area
Hemodialysis	\\
Young patients	Aesthetic defect, contact sports forbidden
Ion channelopathies	Polymorphic or monomorphic VTs not treatable
Hypertrophic cardiomyopathy	Increased risk of T oversensing
Dilated cardiomyopathy	Usually manifested with VTs of variable cardiac frequency (slower VTs not treated by the device)

## Data Availability

Not applicable.
